# Lipid Emulsion Type and Liver Function in Parenteral Nutrition Patients: A Retrospective Study of Patients and Prescribing Practices

**DOI:** 10.3390/nu16162590

**Published:** 2024-08-06

**Authors:** Marvick Melendez, Ronelle Mitchell, Hannah Heredia, Jennifer Lloyd, Jill Taliaferro, Erin K. Beveridge, Stephen J. Ives

**Affiliations:** 1 Optum Home Infusion Services, 8509 Benjamin Rd., Tampa, FL 33364, USA; ronellemitchell@msn.com (R.M.); hwelch@optum.com (H.H.); jennifer_lloyd@optum.com (J.L.); jill.taliaferro@optum.com (J.T.); ebeveridge@optum.com (E.K.B.); 2 Health & Human Physiological Sciences, Skidmore College, Saratoga Springs, NY 12866, USA

**Keywords:** parenteral nutrition, liver function tests, intravenous lipid emulsions

## Abstract

Parenteral nutrition (PN) is a life-sustaining method to provide adequate nutrients to patients unable to receive oral or enteral nutrition. PN typically contains a mixture of macro- and micro-nutrients, although the lipid composition has been identified as a concern for liver disease. Therefore, the study of the intravenous lipid emulsion (ILE) prescribing practices in home-based PN (HPN) patients and whether differing lipid PN alters liver function tests (LFTs) is needed. Methods: A retrospective study of monthly LFTs from a random sample of 105 adult HPN patients in the U.S. over a 6-month period was conducted. Patients were receiving olive oil/soy oil (n = 53, Clinolipid), mixed ILE (n = 39, SMOF Lipid), soy oil (SO; n = 4, Intralipid), or none (n = 7). LFTs monitored were alkaline phosphatase (ALP), alanine transaminase (ALT), aspartate transaminase (AST), and total bilirubin (T Bili). Results: No differences were observed in baseline LFTs across groups (all, *p* > 0.25, η^2^ < 0.04), nor were there differences in age, body mass index, days of PN, or mean PN volume (all, *p* > 0.36, η^2^ < 0.05). There were no significant interactions between ILE type and time (all *p* > 0.64, η_p_^2^ < 0.03), no effect of ILE type (all *p* > 0.60, η_p_^2^ < 0.03), and no effect of time (all *p* > 0.69, η_p_^2^ < 0.01) in terms of LFTs. Average LFTs over six months were also not different between ILE types (all *p* > 0.30, η^2^ < 0.04). Conclusion: These findings suggested that patients were mostly prescribed mixed or ILE PN containing more than one lipid source and that differing ILEs in long-term HPN patients did not alter LFTs over a six-month period.

## 1. Introduction

Parenteral nutrition (PN) is a life-sustaining form of intravenous nutrition used for patients with varying degrees of intestinal failure and patients unable to receive adequate oral or enteral nutrition [1,2]. However, some reports suggest that survival in patients receiving PN ranges from 58% [3] to upwards of 86% [4] in long-term PN patients, which suggests that while a life-sustaining intervention, PN can be further optimized. One consequence of long-term PN is intestinal failure-associated liver disease (IFALD), whose etiology remains multifactorial but increases the risk of mortality [1,2,5]. The type of intravenous lipid emulsion (ILE) is purported to be a risk factor for intestinal failure-associated liver disease (IFALD) [6,7,8]. Even though the exact origin of IFALD is elusive, the subsequent risk of mortality remains a major cause for concern [5]. ILEs are a source of concentrated energy that provides essential fatty acids (EFA) that cannot be synthesized by humans requiring an exogenous source and should not be omitted from the PN regimen [2,7,9]. Clinicians may reduce or remove ILEs when LFTs trend upward, thereby increasing the risk of essential fatty acid deficiency (EFAD) due to patients not receiving an adequate dose of EFAs. The omission of lipids may prompt an increase in dextrose needed to maintain caloric requirement, which can lead to steatosis [2,10]. Thus, understanding the relationship between ILE and LFTs is crucial for the effective clinical management of patients using PN.

With multiple ILE products available and ongoing supply shortages [11], there is a significant disparity between the cost of goods and available reimbursement, and clinicians need to have current evidence to compare the clinical outcomes of the differing ILE formulations. Product reimbursement often falls short of covering the higher cost of goods, rendering some ingredients inaccessible. Therefore, it is crucial to review current ILE products and clinical practice, as current literature lacks comparisons of differing formulations over time in patients receiving ILE PN.

The primary goal of this retrospective cohort study was to first characterize the use and type of ILE in home-based infusion service patients and, second, to compare the liver function tests according to the type of ILE infused in long-term adult HPN patients over a six-month period. A secondary goal was to determine if other clinical parameters, such as HPN calorie provision, medical diagnosis, presence of gall bladder, and/or incidence of a bloodstream infection, played a role in liver function tests.

## 2. Methods

A retrospective study of 105 randomly sampled adult HPN patients on service with a national HPN company from November 2022 to May 2023 were reviewed. Inclusion criteria consisted of adult long-term patients (>18 years old), actively receiving HPN ≥3 days per week, and patients who had routine lab results available. Exclusion criteria included patients receiving HPN < 6-month duration, were non-adherent to therapy, or a lack of available lab results. Data within an electronic medical record were reviewed for clinical parameters, including sex, age, BMI, medical diagnosis, weight, mean PN days, and calories per day provided by HPN. All cases were de-identified prior to aggregation and analysis. This study was reviewed and approved under exempt status by our local Institutional Review Board at United Health Group (IRB#2024-0011, approved 1 February 2024).

The medical diagnoses were grouped into 8 categories based on the medical condition that precipitated the need for PN: small bowel obstruction, inflammatory bowel disease, short bowel syndrome (SBS), enterocutaneous fistula (ECF), gastric bypass complications (GBC), cancer, dysmotility, and malabsorption. Some medical conditions may have overlapped; therefore, patients were grouped into the primary diagnosis for PN. Adult patients grouped with SBS had an inadequate absorptive capacity due to a decrease in bowel length or bowel function. Patients in the malabsorption group included individuals requiring PN secondary to pancreatitis, pancreatic insufficiency, or transplant. Patients in the gastric bypass group experienced a surgical complication from the gastric procedure, which necessitated TPN. Individuals in the inflammatory bowel disease group had a compromised ability to tolerate an oral diet secondary to Crohn’s or ulcerative colitis. Patients in the ECF category may also have had an underlying diagnosis of an inflammatory condition but were grouped separately, as their ECF precipitated the need for PN.

Based on the random sample, there were four groups, receiving either mixed ILE consisting of soy, medium-chain triglyceride (MCT), olive oil, and fish (SMOF Lipid, Fresenius Kabi, Lake Zurich, IL, USA), 80% soy oil (SO) and 20% olive oil (OO; Clinolipid, Baxter, Deerfield, IL, USA), 20% SO (Intralipid, Fresenius Kabi, Lake Zurich, IL, USA), or no ILE. The fatty acid profile of SMOF Lipid is as follows: 23–35% oleic, 14–25% linoleic, 13–24% caprylic, 7–12% palmitic, 5–15% capric, 1.5–4% stearic, 1.5–3.5% alpha-linolenic, 1–3.5% eicosapentaenoic, and 1–3.5% docosahaexaenoic acids. The fatty acid profile of Clinolipid is as follows: 14–22% linoleic, 44–79.5% oleic, 7.6–19.3% palmitic, 0.5–4.2 linolenic, 0.0–3.2% palmitoleic, and 0.7–5% stearic acids. The fatty acid profile of Intralipid is as follows: 44–62% linoleic, 19–30% oleic, 7–14% palmitic, and 4–11% alpha-linolenic acids. We isolated other variables, including sex, age, BMI, mean PN days, mean PN volume, mean lipid gm/kg, and mean kcal/kg, to infused ILE. For the 3 treatment arms receiving ILE, patients all received an average of 0.8 g/kg dose (see Table 1). Labs were reviewed for the following: alkaline phosphatase (ALP), alanine transaminase (ALT), aspartate transaminase (AST), and total bilirubin (T Bili). The lab results were reviewed based on the patient’s lab draw frequency, and a monthly average was derived for ALP, AST, ALT, and T Bili.

## 3. Statistical Analysis

All data were analyzed using open-source software (JASP, v. 18.0, Amsterdam, The Netherlands). The distribution of nominal patient characteristics (e.g., indication for PN) across ILE groups were assessed using contingency tables and Chi^2^ analysis. Potential differences in baseline characteristics between groups, and thus consideration as a covariate in the analysis, were assessed using one-way analysis of variance (ANOVA) and the eta^2^ model of effect size (η^2^, 0.01, 0.06, and 0.14, as small, medium, or large effects, respectively). If a characteristic, such as age, was found to be significant, it would be included as a covariate in the model below. Pairwise comparisons were performed using independent samples *t*-tests and Cohen’s d model of effect size (0.2, 0.5, and 0.8, as small, medium, or large effects, respectively). To explore the potential for whether ILE type may alter LFTs over time, two-way ANOVA and the partial eta^2^ (η_p_^2^, 0.01, 0.06, and 0.14, as small, medium, or large effects, respectively) model of effect size were used. In this design (F-test) with α = 0.05, to test the interaction of groups over time (within–between interaction), a total sample size of 28 would be needed to adequately power (0.8) the detection of significance with a medium (f = 0.25) effect size (G*power, Dusseldorf, Germany). Focusing on the group (between) effect in the repeated-measures ANOVA, using the same parameters as above, a total of 108 patients would be needed to detect a significant difference. Tests of normality (Shapiro–Wilk) and assumptions were performed, appropriate for the model (e.g., Levene’s equality of variance), and if a significant violation was found, an appropriate adjustment was made to the degrees of freedom (e.g., Welch in the ANOVA) or a non-parametric alternative test was employed (e.g., Mann–Whitney U test). Significant main effects were followed up using Tukey’s honestly significant difference post hoc pairwise comparisons. To understand the potential relations amongst the LFTs and how the baseline may relate to values at the end of the observation period, we conducted a correlational analysis and generated a correlation matrix. Again, the assumptions for this test were assessed using the Shapiro–Wilk test of bivariate normality, and if violations were found, the Spearman’s Rho test was used. Alpha was set at 0.05. Data are presented as means ± standard deviation, unless noted otherwise.

## 4. Results

A total of 105 adult patients selected randomly across the United States were reviewed. The distributions of ILEs prescribed are outlined in Table 1. Here, 71% of patients were female (n = 75) and 29% were male (n = 30). The median age of patients was 52.9 ± 15.8 years. The youngest patients within our study group primarily had a dysmotility diagnosis and had an average age of 43 years old. BMI was highest in patients with GBC and ECF (see Table 1 and Table 2). The most common diagnoses for TPN were SBS (35%), dysmotility (20%), and malabsorption (12%; Table 1). SBS affected both males and females, while dysmotility predominately affected females (77%).

LFTs by medical condition had no significant differences in AST (*p* = 0.23, η^2^ = 0.21), ALT (*p* = 0.29, η^2^ = 0.14), ALP (*p* = 0.09, η^2^ = 0.23), or TBili (*p* = 0.19, η^2^ = 0.15). Although, a notable difference was seen in ALP for patients diagnosed with IBD, ECF, malabsorption, and cancer, which are all inflammatory in nature. When reviewing the type of ILE received, most patients received OO/SO ILE totaling 50% (Clinolipid, n = 53), followed by mixed ILE for 39% of patients (SMOF Lipid, n = 41) and a small group of patients receiving 100% SO ILE (Intralipid, n = 4). Isolating the clinical indication for TPN and infused ILE, most patients with SBS received mixed ILE 49% (n = 37), followed by 38% (n = 18) SO/OO. The second most common indication for TPN was dysmotility, and 66% of patients received SO/OO (n = 14), followed by 24% mixed ILE (n = 5). See Table 1 for a complete breakdown of TPN indication by ILE type. The average grams of ILE per day was highest in patients receiving SO/OO averaging 0.81 g/kg and mixed ILE averaging 0.79 g/kg. The medical conditions receiving higher doses of ILE per day were malabsorption, SBO, and inflammatory disease (see Table 2). The doses of ILE (*p* = 0.63, η^2^ = 0.01), PN volume (*p* = 0.22, η^2^ = 0.11), relative caloric intake (*p* = 0.62, η^2^ = 0.07), and BMI (*p* = 0.17, η^2^ = 0.12) were not different across indication for PN, only age was (*p* = 0.02, η^2^ = 0.19).

Baseline LFT did not show significant differences between patients with and without a gallbladder. Specifically, levels of AST (39.7± 38.7 vs. 31.0 ± 22.6 U/L, *p* = 0.18, d = 0.3), ALT (44.4 ± 29.0 vs. 39.6 ± 48.1 U/L, *p* = 0.62, d = 0.1), or TBili (0.58 ± 0.61 vs. 0.49 ± 0.43 mg/dL, *p* = 0.17, d = 0.2) were similar between both groups. However, ALP levels were higher in those without a gall bladder (198.4 ± 240 vs. 131.5 ± 85.6 U/L, *p* = 0.05, d = 0.4).

There was no significant difference in baseline LFTs in patients without compared to those with a bloodstream infection in the last 1000 days. LFTS were not significantly different for AST (32.4± 21.5 vs. 34.9 ± 43.3 U/L, *p* = 0.71, d = 0.1), ALT (42.2 ± 44.9 vs. 34.0± 31.5 U/L, *p* = 0.40, d = 0.2), ALP (142.7 ± 97.1 vs. 180.5 ± 253.6 U/L, *p* = 0.29, d = 0.2), or TBili (0.48 ± 0.39 vs. 0.60 ± 0.71 mg/dL, *p* = 0.33, d = 0.2). Additionally, 96% of patients (n = 101) were allowed PO intake. LFTs were not significantly different by PO intake (all *p* > 0.05, d < 0.5), but AST (41.8 ± 31.1 vs. 32.1 ± 27.3 U/L), ALP (431.7 ± 598.8 vs. 138.6 ± 89.3 U/L), and TBili (0.98 ± 1.4 vs. 0.48 ± 0.40 mg/dL) tended to be higher in those not allowed PO, but not ALT (33.7 ± 19.9 vs. 39.4 ± 41.3 U/L).

### Potential Impact of ILE Type on LFTs

**Figure 1:**
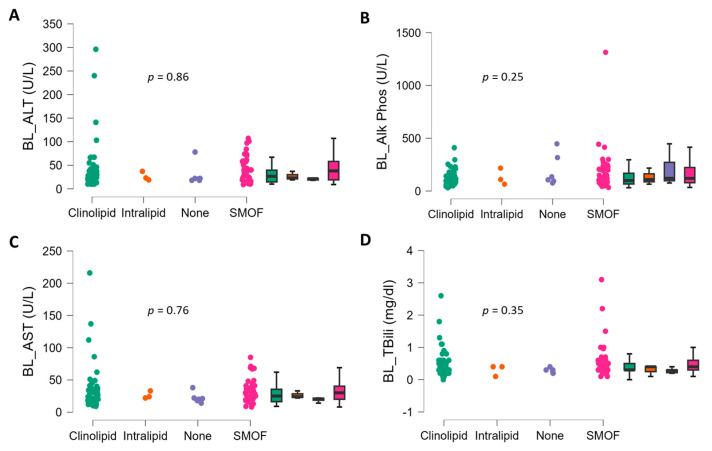
Baseline LFTs in adult home PN patients (n = 105) according to ILE type. (**A**) Alanine transaminase (ALT), (**B**) alkaline phosphatase (ALP), (**C**) aspartate transaminase (AST), and (**D**) total bilirubin (TBili). Data are individual points and box and whisker plots (median and IQR). The *p* values for group comparison were from analysis of variance.

There were no significant differences in baseline age (*p* = 0.87, d = 0.0), number of days of PN (*p* = 0.37, d = 0.0), PN volume (*p* = 0.78, d = 0.0), or BMI (*p* = 0.48, d = 0.0) between ILE subject groups. There were also no differences in sex (*p* = 0.44), indication for PN (*p* = 0.59), recent bloodstream infection (*p* = 0.18), PO allowed (*p* = 0.72), incidence of diabetes (*p* = 0.68), or gall bladder removal (*p* = 0.16) across the ILE treatment groups. No significant differences were observed in baseline AST (*p* = 0.76, η^2^ = 0.01), ALT (*p* = 0.86, η^2^ = 0.01), ALP (*p* = 0.25, η^2^ = 0.04), and TBili (*p* = 0.35, η^2^ = 0.04) across ILE treatment groups (Figure 1).

**Figure 2:**
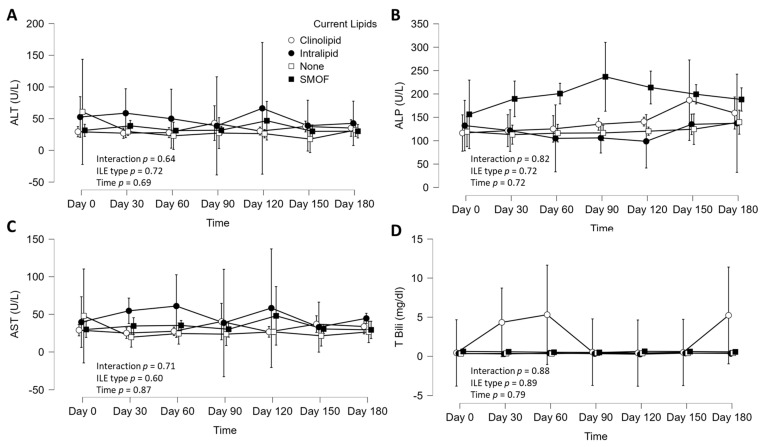
LFTs in adult home PN patients (n = 105) according to ILE type over time. (**A**) Alanine transaminase (ALT), (**B**) alkaline phosphatase (ALP), (**C**) aspartate transaminase (AST), (**D**) and total bilirubin (TBili). Data are mean ± 95% confidence intervals. The *p* values are for group comparison over time using two-way analysis of variance.

**Figure 3:**
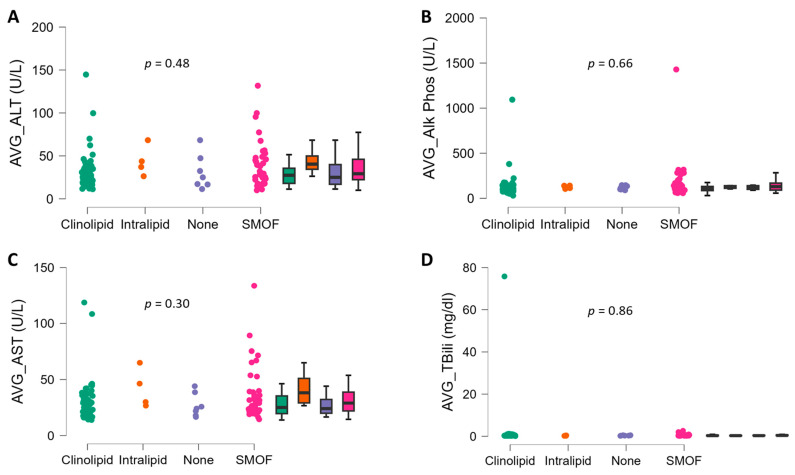
The average of LFTs in adult home PN patients (n = 105) according to ILE type over the 6-month observation period. (**A**) Alanine transaminase (ALT), (**B**) alkaline phosphatase (ALP), (**C**) aspartate transaminase (AST), and (**D**) total bilirubin (TBili). Data are individual points and box and whisker plots (median and IQR). The *p* values for group comparison were from analysis of variance.

There were no significant interactions between ILE type and time (six-month observation) for AST (*p* = 0.71, η_p_^2^ = 0.03), ALT (*p* = 0.64, η_p_^2^ = 0.03), ALP (*p* = 0.82, η_p_^2^ = 0.02), and TBili (*p* = 0.88, η_p_^2^ = 0.02; Figure 2). There were no main effects of ILE treatment type for AST (*p* = 0.60, η_p_^2^ = 0.03), ALT (*p* = 0.72, η_p_^2^ = 0.02), ALP (*p* = 0.72, η_p_^2^ = 0.02), or T. Bili (*p* = 0.89, η_p_^2^ = 0.01; Figure 2). There were also no main effects of time for AST (*p* = 0.87, η_p_^2^ = 0.00), ALT (*p* = 0.69, η_p_^2^ = 0.01), ALP (*p* = 0.72, η_p_^2^ = 0.00), or TBili (*p* = 0.79, η_p_^2^ = 0.00; Figure 2). The averages of the LFTs over six months also were not different between ILE type for AST (*p* = 0.30, η^2^ = 0.04), ALT (*p* = 0.48, η^2^ = 0.02), ALP (*p* = 0.66, η^2^ = 0.02), or TBili (*p* = 0.85, η^2^ = 0.01; Figure 3). Finally, body weight did not differ by ILE type (group, *p* = 0.43, η^2^ = 0.03), or by ILE type over time (interaction, *p* = 0.79, η^2^ = 0.01). However, there was a significant time effect (time, *p* = 0.00, η^2^ = 0.00), where body weight increased, on average, by 5 kg (60.3 ± 9.8 to 65.5 ± 9.8 kg).

**Figure 4:**
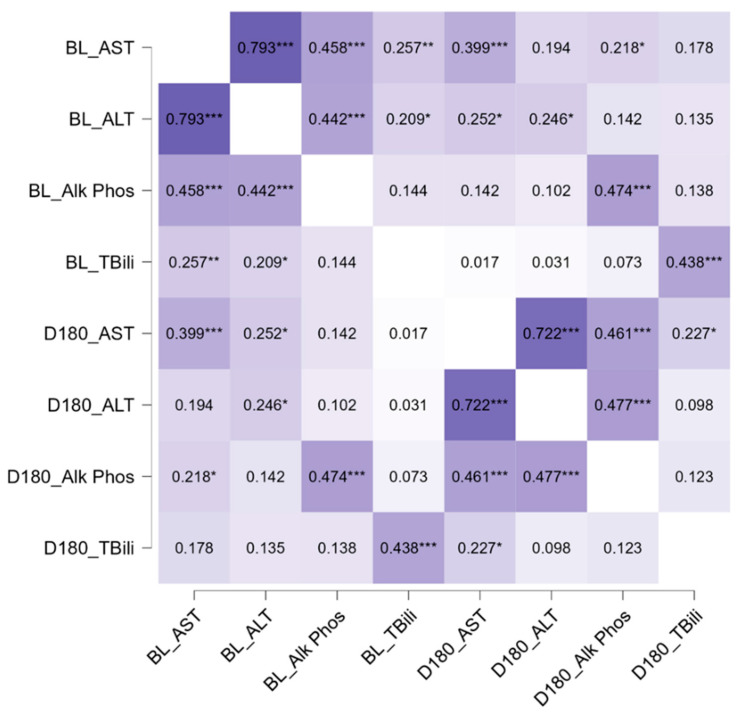
Correlation matrix amongst the LFTs in adult home PN patients (n = 105) from baseline to the end of the six-month observation period. Alanine transaminase (ALT), aspartate transaminase (AST), alkaline phosphatase (ALP), and total bilirubin (TBili). Data are Spearman’s Rho correlation coefficients; * *p* < 0.05, ** *p* < 0.01, and *** *p* < 0.001 for each bivariate relation.

To better understand the potential relation among the different liver function tests (AST, ALP, ALT, and TBili) and how baseline LFT values related to values obtained at the end of the study observation window, we employed correlational analysis. The correlation matrix is presented in Figure 4 below. Briefly, all four LFTs were significantly associated with one another (all ρ > 0.26, *p* < 0.01, Figure 4), with the strongest association between ALT and AST (ρ = 0.79, *p* < 0.001, Figure 4). When exploring the relation between baseline and end-study LFTs, the individual baseline LFTs were positively associated with the corresponding end-study LFTs (all ρ > 0.25, *p* < 0.05, Figure 4).

## 5. Discussion

We aimed to characterize prescribing practices by exploring the ILE type that HPN patients were receiving and determine if the ILE type prescribed altered liver function tests in patients receiving PN. First, perhaps dictated by market availability and current recommendations, was that predominant ILEs prescribed were ILEs containing more than one fat, with almost rare use of single or no lipids. Second, we found no differences in LFTs by ILE type, time, or their interaction, and attributed this to improved strategies in central venous access device (CVAD) care, close clinical monitoring, and multidisciplinary communication between pharmacists, dietitians, nurses, and prescribing clinicians. We did observe significant relations between the LFTs at baseline and with end-study values, suggesting that an elevated single LFT coalesces with elevation in other LFTs and that this holds true over a six-month period. Further, no significant differences in LFTs were observed with PO intake, the presence of a gall bladder, or whether they had a recent infection within the last 1000 days. Ongoing management, including regular weight checks, helped our dietitians monitor and adjust calorie goals. These findings suggest that clinicians favor mixed-source ILE PN, and that short-term ILE use did not alter liver function and thus perhaps the risk of IFALD, though longer-term real-world studies are needed.

### 5.1. ILE Type, LFTs, and IFALD

Our study found that current prescribing practices predominantly involve mixed-source ILE to mitigate potential adverse effects associated with SO, such as elevated LFTs and cholestasis [12]. Second, the average relative dose of ILE appeared within the recommended dosages, with average dosing of ~0.8 g/kg/d [12]. The current findings largely agree with another previous similar study, which demonstrated no difference between ILE types, the exception being that we did not observe a reduction in TBili in the SMOF group, but this may be due to the shorter observation period [13]. Thus, in a random sample of regionally diverse, long-term HPN patients in the U.S., well-matched demographically across ILE type, we did not find any clear evidence of IFALD or elevated IFALD risk according to ILE TPN type. This insight into clinical practice and outcomes is essential in the ongoing clinical management of TPN patients.

### 5.2. Essential Fatty Acids (EFA) and Potential Infection Role

Providing adequate EFA is necessary, as they play a valuable role, including metabolic support, immunity, and cell wall integrity [14]. Older literature blamed ILE for central-line-associated bloodstream infections (CLABSI); however, improved central venous access device (CVAD) management and compounding, along with avoidance of overfeeding, has helped to decrease CLABSI incidence [15]. Therefore, clinicians should avoid excluding ILE in the PN to minimize CLABSI. In the present study, we did not observe a difference in LFTs between those with recent infection and those without. As noted by Gavin et al. (2023), lipid-free TPN did not reduce the risk of infection or PNALD but postulated the role the gut integrity may have in bacterial translocation subsequent to bloodstream infection. Properly dosed ILEs are not culpable in bloodstream infections; however, a septic event can precipitate consequential liver disease and result in PNALD [16], as endotoxins activate inflammatory cytokines that downregulate transcription, leading to cholestasis and ductal proliferation [17,18].

### 5.3. Phytosterols and IFALD

Phytosterols play a role in liver dysfunction and IFALD [2,19,20]. Phytosterols are compounds similar in structure and function to cholesterol, which are found in plant cell membranes [10]. The most common phytosterols found in ILE formulations are sitosterol, campesterol, and stigmasterol. Studies have suggested phytosterols, especially stigmasterol, which can interfere with bile acid synthesis, hepatobiliary transporters, and nuclear receptors, stimulating proinflammatory cytokines and impacting the risk of IFALD [20,21]. The phytosterol content as documented elsewhere [20], is highlighted below (Table 3). OO ILE contains the lowest levels of phytosterols and SO contains the highest [20,21]. It is thought that ILE containing higher amounts of phytosterols play a role in the risk of IFALD; however, these were not measured in this study. In line with clinical recommendations to minimize pro-inflammatory SO, clinicians predominately favored SO/OO or mixed ILE [22], likely contributing to no apparent differences in LFTs amongst the groups, at least over this six-month observation of these long-term HPN patients.

### 5.4. Role of Demographic Factors in LFTs of Patients Receiving PN

Although not statistically significant, patients without a gallbladder had a tendency for higher LFTs. An extensive literature search resulted in a lack of references available discussing PN in patients without a gallbladder and its effects on LFTs. Out of a total of 105 patients in this study, 33 did not have a gallbladder present and had higher, on average, LFTs, including AST, ALT, ALP, and TBili. This may suggest that having a gallbladder may offer some protection in the development of intestinal-failure-associated liver disease (IFALD). Future research is indicated. Similarly, whether patients were allowed PO intake also influenced LFTs, whereas those not allowed tended to have higher LFTs. The medical indication for PN did not appear to significantly alter LFTs, but the circulating biomarkers did tend to vary by condition. The dose of ILE, volume of PN, relative caloric dosing, and BMI were not different across indication for PN; however, age was different according to medical indication for PN. Collectively, while many of these patient demographic factors were not statistically significant, they may be important in the individual clinical management of these patients.

### 5.5. Strengths and Limitations of the Current Study

A strength of the study was the random selection of regionally diverse patients across seven states, including Texas, Arizona, New Jersey, Florida, Nebraska, and New York, active during our selected time period. Another strength of the present study is that among this random sample, the HPN patients with differing ILE prescriptions were relatively well matched across groups on factors that could influence LFTs, such as recent infection, indication for PN, presence of gall bladder, age, sex, BMI, as well as frequency and volume of PN. While six months is relatively robust and perhaps possible to see elevated LFTs, we considered it a limitation to our study and recommend a longer time span to capture changes. However, it is important to note that the average length on the service for the patients within this retrospective review was 3 years overall and up to 5.3 years for patients with SBS. Further research is recommended to explore a longer duration of lab review among HPN patients. Another limitation of our study was an inability to track circulating essential fatty acids across ILE groups to rule out if EFAD may have been present in patients with lab abnormalities. Future studies should explore the potential genetic, epigenetic, and physiological mechanisms that underpin the susceptibility to IFALD. Further, future studies should explore if functional outcomes, such as hepatosteatosis, differ according to ILE type prescribed.

## 6. Conclusions

The pathophysiology of IFALD is not completely understood, but likely multifactorial and long-term ecological studies of patients and ILE-prescribing practices are needed. Our six-month study of long-term HPN patients revealed that ILE type did not affect LFTs across groups that were demographically well matched and consisted of mostly mixed ILE or SO/OO with an average PN infusion of six days/week. LFTs, although non-significant, were highest among inflammatory conditions, and those without a gallbladder. These findings suggest that different ILE prescriptions in HPN patients do not alter typical LFTs over the short term, and that optimizing management through maintaining the ILE dose (<1 g/kg/day) [12,22], minimizing overfeeding, risk of infection, and promoting gut mucosal integrity may be beneficial toward reducing IFALD.

## Figures and Tables

**Table 1:** Patient demographics by type of intravenous lipid emulsion (ILE) the patients were prescribed.

Demographics	Intralipid	Clinolipid	SMOF Lipid	No Lipids
Female/Male (n)	4/0	36/17	29/12	6/1
Age (years)	55.8 ± 11	52.1 ± 16	53.1 ± 17	56.9 ± 9
BMI (kg/m^2^)	20.5 ± 2.2	23.9 ± 6.6	21.8 ± 6.9	23.0 ± 5.8
**Nutritional Support**				
Mean PN days (d)	5 ± 2.0	5.9 ± 1.7	6.1 ± 1.3	4.8 ±1.9
Mean PN volume (mL)	1925 ± 431	1836 ± 479	1935 ± 851	1685 ± 623
Mean lipid g/kg/d	0.81 + 0.21	0.78 + 0.25	0.8 + 0.35	n/a
Mean kcal/kg/d	25.2 ± 8.3	22.3 ± 7.9	31.7 ± 6.7	12.2 ± 7.1
**PN Indication**				
Short bowel syndrome (SBS)	1	14	18	4
Inflammatory bowel disease (IBD)	0	4	4	1
Short bowel obstruction (SBO)	0	4	0	0
Dysmotility (DYS)	0	14	5	2
Enterocutaneous fistula (ECF)	0	4	3	0
Gastric bypass complications (GBC)	2	1	3	0
Malabsorption (MA)	1	9	3	0
Cancer	0	3	5	0
**Baseline LFTs**				
AST (U/L, 10–40 NR)	27.3 ± 5.1	33.7 ± 34.9	32.8 ± 18.0	24.1 ± 9.2
ALT (U/L, 6–44 NR)	26.7 ± 7.8	40.0 ± 51.7	40.7 ± 26.7	31.0 ± 21.8
ALP (U/L, 35–153 NR)	136 ± 65	122 ± 75	181 ± 206	185 ± 141
TBili (mg/dL, 0.2–1.2 NR)	0.28 ± 0.15	0.47± 0.44	0.58 ± 0.55	0.30 ± 0.08

Data are frequencies or counts, or mean ± SD. Note: BMI, body mass index; PN, parenteral nutrition; LFT, liver function test; AST, aspartate transaminase; ALT, alanine transaminase; ALP, alkaline phosphatase; TBili, total bilirubin; NR, normal range.

**Table 2:** Patient demographics by PN indication.

	SBS	MA	GBC	DYS	Cancer	SBO	ECF	IBD
**Demographics**								
Age (years)	53.7 ± 16.3	53.2 ± 14.9	49.5 ± 9.7	43.2 ±17.2	70.3± 9.3	47.0 ± 6.0	57.4 ± 9.5	58 ± 12.9
Length of therapy (years)	5.3 ± 3.5	3.1 ± 1.8	2.1 ± 5.4	4.3 ± 2.6	1.4 ± 1.2	2.3 ± 1.7	2.5 ± 2.6	2.7 ± 2.3
Avg kcal/kg/d	24.1 ± 9.1	26.3 ± 7.6	22.9 ± 13.8	17.2 ± 7.5	24.9 ± 8.4	25.8 ± 9.3	21.2 ± 5.6	23.9 ± 7.5
ILE g/kg/d	0.8 ± 0.4	0.9 ± 0.2	0.6 ± 0.4	0.8 ± 0.4	0.8 ± 0.2	0.9 ± 0.5	0.7 ± 0.3	0.9 ± 0.4
BMI (kg/m^2^)	22.9 ± 5.4	19.9 ± 2.8	26.0 ± 9.7	25.4 ± 8.9	22.4 ± 7.3	19.8 ± 5.6	27.9 ± 5.7	23.1 ± 4.9
**LFTs**								
AST (U/L, 10–40 NR)	33.4 ± 23.8	23.8 ± 33.5	35 ± 16.9	28 ± 16.7	38 ± 16.2	32 ± 10.2	28 ± 13.5	33 ± 17.8
ALT (U/L, 6–44 NR)	32 ± 24.8	42 ± 39.9	37 ± 19.2	26 ± 10.9	35 ± 9.6	37 ± 19.5	40 ± 30.1	44 ± 32.9
ALP (U/L, 35–153 NR)	126 ± 47.1	205 ± 251	100 ± 25.6	92 ± 28.8	317 ± 170.1	93 ± 35.7	205 ± 93.1	152 ± 96.0
Tbili (mg/dL, 0.2–1.2 NR)	0.61 ± 0.5	0.39 ± 0.3	0.34 ± 0.1	0.72 ± 12.5	0.5 ± 0.4	0.75 ± 0.5	0.4 ± 0.2	0.33 ± 0.1

Data are mean ± SD. Note: PN, parenteral nutrition; SBS, short bowel syndrome; MA, malabsorption; GBC, gastric bypass complications; DYS, dysmotility; SBO, short bowel obstruction; ECF, enterocutaneous fistula; IBD, inflammatory bowel disease; BMI, body mass index; NR, normal range; AST, aspartate transaminase; ALT, alanine transaminase; ALP, alkaline phosphatase; TBili, total bilirubin.

**Table 3:** Phytosterol content of ILE types in the study (from a prior study [20]).

ILE	Sitosterol (µg/mL)	Campesterol (µg/mL)	Stigmasterol (µg/mL)	Total Phytosterol Content (µg/mL)
Intralipid (SO)	302.6 ± 2.0	55.4 ± 0.5	65.1 ± 0.5	439.1 ± 5.7
Clinolipid (OO and SO)	240.6 ± 2.1	13.3 ± 0.1	12.2 ± 0.0	274.4 ± 2.6
SMOF Lipids (SO, MCT, OO, and FO)	131.6 ± 7.1	20.5 ± 1.0	18.5 ± 0.8	178.5 ± 9.6

Note: Data are mean ± SD.

## Data Availability

The data are available upon reasonable request to the authors.
